# Squalene Cyclases and Cycloartenol Synthases from *Polystichum polyblepharum* and Six Allied Ferns

**DOI:** 10.3390/molecules23081843

**Published:** 2018-07-24

**Authors:** Junichi Shinozaki, Takahisa Nakene, Akihito Takano

**Affiliations:** Faculty of Pharmaceutical Sciences, Showa Pharmaceutical University, Machida, Tokyo 194-8543, Japan; nakane@ac.shoyaku.ac.jp (T.N.); takano@ac.shoyaku.ac.jp (A.T.)

**Keywords:** squalene cyclase, cycloartenol synthase, triterpene, fern, *Polystichum*

## Abstract

Ferns are the most primitive of all vascular plants. One of the characteristics distinguishing them from flowering plants is its triterpene metabolism. Most cyclic triterpenes in ferns are hydrocarbons derived from the direct cyclization of squalene by squalene cyclases (SCs). Both ferns and more complex plants share sterols and biosynthetic enzymes, such as cycloartenol synthases (CASs). *Polystichum* belongs to Dryopteridaceae, and is one of the most species-rich of all fern genera. Several *Polystichum* ferns in Japan are classified as one of three possible chemotypes, based on their triterpene profiles. In this study, we describe the molecular cloning and functional characterization of cDNAs encoding a SC (PPH) and a CAS (PPX) from the type species *Polystichum polyblepharum.* Heterologous expression in *Pichia pastoris* revealed that PPH and PPX are hydroxyhopane synthase and CAS, respectively. By using the PPH and PPX sequences, we successfully isolated SC- and CAS-encoding cDNAs from six *Polystichum* ferns. Phylogenetic analysis, based on SCs and oxidosqualene cyclase sequences, suggested that the *Polystichum* subclade in the fern SC and CAS clades reflects the chemotype—but not the molecular phylogeny constructed using plastid molecular markers. These results show a possible relation between triterpenes and their biosynthetic enzymes in *Polystichum.*

## 1. Introduction

Ferns are the most primitive of all vascular plants. They produce specialized characteristic metabolites that are not found in flowering plants [1]. These include triterpenoids and phloroglucinols with various biological activities [2,3,4]. Fern triterpenoids are biosynthesized in a manner that is significantly different from those in flowering plants. Most ferns produce triterpene hydrocarbons by the cyclization of squalene, a C30 acyclic substrate (**1**). Their physiological function remains largely elusive. The enzymes responsible for these reactions are squalene cyclases (SCs). Fern SCs are homologous to those in bacteria. While bacterial SCs generate hop-22(29)-ene (**2**) [5,6,7,8,9,10], fern SCs (discovered thus far) yield various cyclic triterpenes, such as migrated hopane and germanicane (pentacycles), dammarane and tirucallane (tetracycles), bicyclic polypodane skeletons, and hopane skeletons (**2**) [11,12,13,14]. However, several fern phytosterols are identical to those found in flowering plants. At the gene/enzyme level, the sterol pathway might be conserved between ferns and flowering plants. Cycloartenol synthases (CASs), which are oxidosqualene cyclases (OSCs) involved in sterol metabolism, have been successfully cloned from ferns [11,13]. Because ferns lie between bacteria and flowering plants on the evolutionary scale, they produce triterpenes that are similar to those produced by bacteria and flowering plants. Therefore, the study of ferns might be useful in investigating the molecular evolution of triterpene biosynthesis from bacterial to flowering plants.

**Figure 1:**
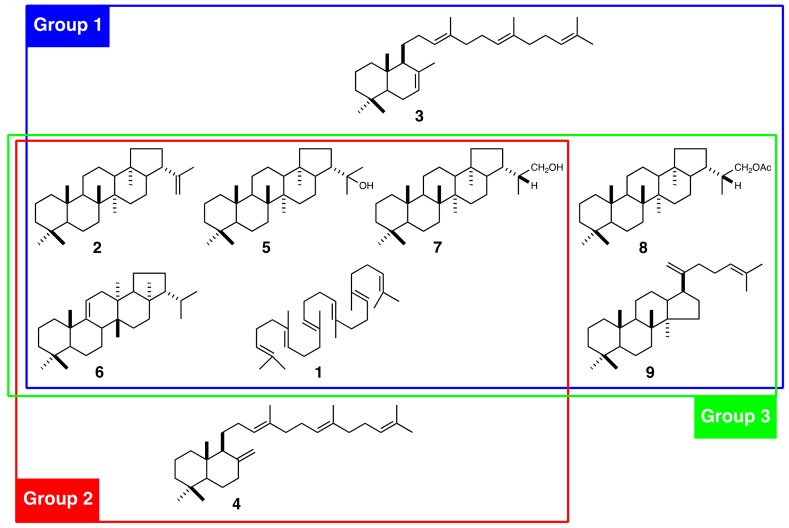
Triterpenoids isolated from *Polystichum* ferns and chemotaxonomic classification based on the triterpenoid profile. Group 1 includes *P. polyblepharum*, *P. fibrilloso-paleaceum*, and *P. pseudo-makinoi*; Group 2 includes *P. rigens*; and Group 3 includes *P. longifrons*, *P. makinoi*, and *P. lepidocaulon*. **1**, squalene; **2**, hop-22(29)-ene; **3**, γ-polypodatetraene; **4**, α-polypodatetraene; **5**, 22-hydroxyhopane; **6**, fern-9(11)-ene; **7**, dryocrassol; **8**, dryocrassyl acetate; and **9**, dammara-18(28),21-diene.

*Polystichum* Roth (Dryopteridaceae) is one of the most species-rich of all fern genera. It is distributed in both temperate and subtropical regions and is found in lowlands, montane, and alpine areas. About >200 *Polystichum* species are estimated to occur worldwide [15]. There are 32 known species in Japan [16], which is one of the most species-rich of all areas. *Polystichum* species have diverse morphological traits and produce several different triterpenoids. These include the bicyclic γ- and α-polypodatetraenes (**3**, **4**) which were the first bicyclic triterpenes identified [17]. The major constituents are pentacyclic hopane and migrated hopane triterpenoids such as compound **2**, 22-hydroxyhopane (**5**), fern-9(11)-ene (**6**), dryocrassol (**7**), and dryocrassyl acetate (**8**) (Figure 1) [18]. The fern triterpenoids have been classified into three chemotaxonomic groups based on the profiles of 16 Japanese *Polystichum* species [19]: Group 1 includes *P. polyblepharum*, *P. fibrilloso-paleaceum*, and *P. pseudo-makinoi.* These produce compound **3** (Figure 1). Group 2 includes *P. rigens*, which produces compound **4**. Group 3 includes *P. longifrons*, *P. makinoi*, and *P. lepidocaulon.* None of these produces either **3** or **4**.

To elucidate the molecular basis of triterpenoid biosynthesis, we cloned SC and OSC from *P. polyblepharum*, the type species, and functionally analyzed their cDNAs by heterologous expression in yeast. By using these results, we attempted to isolate triterpene synthases from six other *Polystichum* species to determine whether molecular phylogeny based on triterpene synthase sequences was correlated with their chemotaxonomic classification.

## 2. Results

### 2.1. Isolation of cDNAs Encoding SC and OSC

We cloned full-length cDNAs from *P. polyblepharum* encoding SC and OSC by using a combination of homology-based PCR and rapid amplification of cDNA ends (RACE). A DNA fragment encoding the putative SC was obtained by reverse-transcription PCR (RT-PCR) by using the degenerate primers SC-306S-1, SC-306S-2, SC-358S, SC-494A, and SC-537A [11,12] targeting highly conserved consensus sequences of bacterial SCs. The PCR product with the expected size of 450 bp was purified using gel electrophoresis, cloned, and amplified in *Escherichia coli*. Sixteen plasmids with inserts of the expected length were extracted from the *E. coli* transformants and sequenced. They were all identical and showed a high sequence identity to known fern SCs. The remaining sequences of the putative SC cDNA were acquired using 5′- and 3′-RACE PCR by using the gene-specific primers designed from the sequence obtained. The full-length cDNA was designated PPH. It consisted of 2530 bp with a 2058 bp coding region, 32 bp 5′ untranslated region (UTR), and 365 bp 3′ UTR region. The open reading frame (ORF) of PPH was a polypeptide 685 residues long with an estimated molecular weight of 77.1 kDa. The amino acid sequence of PPH showed 65–90% and 35–41% identity with functionally characterized SCs from ferns and bacteria, respectively. It most closely matched a hydroxyhopane synthase from Adiantum capillus-veneris (ACH).

A nested PCR was run with two sets of degenerate primers (OSC-162S, OSC-463S, OSC-467S, OSC-623A, and OSC-711A) [20,21] and cDNA prepared from template RNA. It yielded a 400 bp DNA fragment. A full-length cDNA sequence, including clonal 5′ and 3′ UTR, was designated as PPX. It was obtained using the RACE method by using gene-specific primers derived from the 400 bp DNA fragment. The full-length cDNA of PPX was 2596 bp in length and contained a 76 bp 5′ UTR, 234 bp 3′ UTR, and 2286 bp ORF corresponding to a deduced protein sequence of 761 amino acid residues (86.7 kDa). The amino acid sequence of PPX had a 66–89% identity with the CASs of other plants, including ferns. In contrast, it had relatively low identity (42–62%) with other OSCs. The highest amino acid identity score was 89% for a CAS derived from *Polypodiodes niponica* (PnCAS).

### 2.2. Heterologous Expression and Biochemical Characterization of PPH and PPX

**Figure 2:**
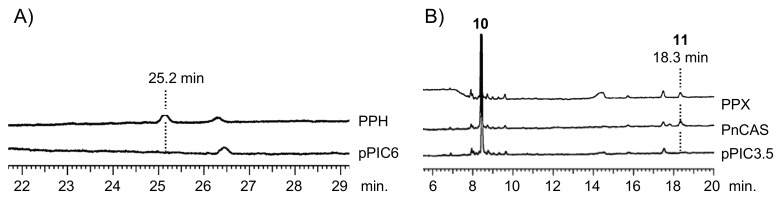
GC analysis of PPH (**A**) and PPX (**B**) products. (**A**) Gas chromatograms of triterpene mono-alcohol fraction from yeast transformants with PPH and pPIC6 (empty vector). Retention time of the PPH product was 25.2 min. (**B**) Gas chromatograms of triterpene mono-alcohol fraction from in vitro reaction of racemic 2,3-oxidosqualene (**10**) with recombinant PPX, CAS derived from *Polypodiodes niponica* (PnCAS), and empty vector (pPIC3.5). Retention time of the PPX and PnCAS product was 18.3 min.

The biochemical functions of PPH and PPX were elucidated by heterologously expressing them in the methylotrophic yeast *Pichia pastoris*. Yeast cells overexpressing PPH generated one product not present in control yeast cells. It carried an empty vector pPIC6 (Figure 2A). The addition of the squalene epoxidase inhibitor terbinafine to a post-induction culture revealed that PPH could use the accumulated endogenous squalene as an in vivo substrate. The PPH product was purified using silica-gel column chromatography and eluted as a hexane-ethyl acetate (100:3, *v*/*v* %) fraction. It did not elute as a hexane fraction. Therefore, it was a triterpene mono-alcohol. It was identified as 22-hydroxyhopane (**5**) by comparing its ^1^H NMR spectrum with that of an authentic standard [22]. The results were highly suggestive that PPH encodes hydroxyhopane synthase in *P. polyblepharum*.

The ORF of PPX was subcloned into the expression vector pPIC3.5. The construct was transformed into *P. pastoris* strain GS115. A cell-free extract prepared from the induced transformant culture was used to test the in vitro reaction. The recombinant PPX cyclized racemic 2,3-oxidosqualene (**10**) to a product not detected in the negative control yeast cell homogenate carrying the empty vector pPIC3.5. Gas chromatography (GC) analysis showed that the hexane extract of the in vitro reaction of PPX was superimposed onto that of PnCAS (Figure 2B) [13]. Therefore, the cDNA sequence of PPX encodes *P. polyblepharum* CAS.

### 2.3. Comparison of Active Site Residues between Hopene and Hydroxyhopane Synthases

**Figure 3:**
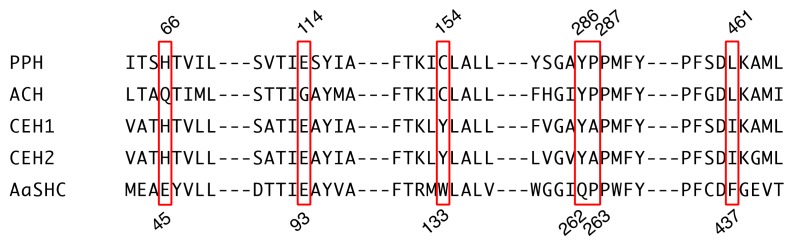
Partial amino acid sequence alignment of hydroxyhopane/hopene synthases. Numbers above the sequence show positions in PPH, and numbers below the sequence show those in AaSHC. Red boxes indicate active site residues, which might influence the final cation elimination reaction.

We compared the active sites of PPH and ACH [11], CEH1 and CEH2 from *Colysis elliptica* (hopene synthases) [14], and AaSHC from *Alicyclobacillus acidocaldarius* (a bacterial hopene synthase; Figure 3). Mutagenesis experiments with AaSHC identified 10 amino acid residues (Thr41, Glu45, Glu93, Arg127, Trp133, Gln262, Pro263, Tyr267, Phe434, and Phe437) responsible for the final cation elimination reaction [23]. Four of them (Thr41, Arg127, Tyr267, and Phe434 in AaSHC) were strictly conserved among all five enzymes (Supplementary Figure S2). Glu93 in AaSHC was also conserved among all enzymes except ACH, which had a Gly at that location (Figure 3). PPH, ACH, and AaSHC had Pro263, but the corresponding residue in the fern hopene synthases (CEH1 and CEH2) was Ala (Figure 3). The following residues were conserved among the fern SCs: Glu45 in AaSHC was replaced by His (Gln in ACH); Gln262 in AaSHC was replaced by Tyr; and Phe437 in AaSHC was replaced by Leu in PPH and ACH and by Ile in CEH1 and CEH2 (Figure 3). Trp133 in AaSHC was not conserved among the fern SCs. The corresponding residues were Cys in PPH and ACH and Tyr in CEH1 and CEH2 (Figure 3).

### 2.4. Phylogenetic Analysis of SCs and OSCs

**Figure 4:**
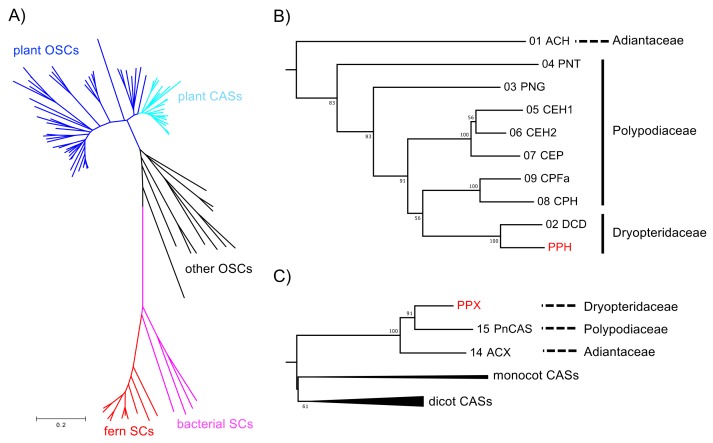
Phylogenetic analysis of PPH and PPX. (**A**) Unrooted neighbor-joining phylogenetic tree of OSC and SC homologs. Plant OSC and CAS clades are in blue and pale blue, respectively. Fern and bacterial SC clades are in red and magenta, respectively. For clarity, the branches have no taxon labels. The tree showing all taxon names is presented in Supplementary Figure S1. The scale represents 0.1 amino acid substitution per site. (**B**) Expanded fern SC clade from the phylogenetic tree in (**A**). Bootstrap values with 1000 replicates are shown at the nodal branches (cutoff value = 50%). The enzyme function and species of the sequences are as follows: ACH, hydroxyhopane synthase from *Adiantum capillus-veneris*; CEH1/2, hopene synthase from *Colysis elliptica*; CEP, α-polypodatetraene synthase; CPFa, fern-9(11)-ene synthase from *C. pothifolia*; CPH, hop-17(21)-ene synthase from *C. pothifolia*; DCD, dammaradiene synthase from *Dryopteris crassirhizoma*; PNT, tiriucalladiene synthase from *Polypodiodes niponica*; PNG, germanicene synthase from *P. niponica*. (**C**) Expanded plant CAS clade from the phylogenetic tree in (**A**). Sequences in monocot and dicot branches are collapsed for clarity. Bootstrap values with 1000 replicates are shown at the nodal branches (cutoff value = 50%). The enzyme function and species of the sequences are as follows: ACX, cycloartenol synthase from *A. capillus-veneris*; PnCAS, cycloartenol synthase from *P. niponica*. The accession numbers, abbreviations, enzyme functions, and species are listed in Supplementary Table S1.

A phylogenetic tree was constructed based on the neighbor-joining method (Figure 4) to elucidate the phylogenetic relationships of the deduced amino acid sequences of PPH and PPX with other known SC and OSC members. The sequences were separated into three divergent clades (such as SC and OSC). In the SC clade, the fern SCs were clearly divided into those for bacteria. The PPH of a hydroxyhopane synthase clustered with DCD (a dammaradiene synthase from *Dryopteris crassirhizoma*), but not with ACH; Figure 4B). The OSC clade consisted of a plant and the remaining eukaryotic OSC subclades. The plant OSC subclade was resolved as a monophyletic family separating into CAS and the other OSC clusters. The fern CASs, including PPX, were grouped together and clearly separated from those of higher plant origin. PPX was found to be closely related to PNX (*Polypodiodes niponica*), but not to ACX (*A. capillus-veneris*).

### 2.5. Phylogenetic Relationships between Polystichum Triterpene Synthases

The SC clade in the phylogenetic tree reflects a taxonomic relationship rather than the molecular evolution of enzyme functions. In this case, the tree topology reconstituted from the SC and/or CAS sequences is expected to reveal a chemotaxonomic relationship based on the triterpenoid profiles of various *Polystichum* ferns. We performed an additional phylogenetic analysis focusing on this relationship. We isolated SC- and CAS-encoding cDNAs from six other *Polystichum* ferns to determine whether the tree topology, based on the SC and/or CAS sequences, reflects a taxonomic relationship. The fern species used were *P. fibrillosopaleaceum*, *P. pseudo-makinoi* (chemotaxonomic group 1), *P. rigens* (chemotaxonomic group 2), *P. longifrons*, *P. makinoi*, and *P. lepidocaulon* (chemotaxonomic group 3).

**Figure 5:**
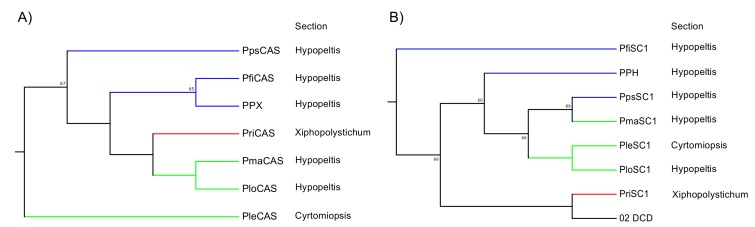
Phylogenetic analysis of *Polystichum* triterpene synthases. (**A**) Expanded *Polystichum* CAS clade. (**B**) Expanded Dryopteridaceae SC clade. Bootstrap values with 1000 replicates are shown at the nodal branches (cutoff value = 50%). Blue, red, and green branches indicate groups 1, 2, and 3, respectively. These are categorized by their triterpene-based chemotaxonomy. DCD is a dammaradiene synthase from *Dryopteris crassirhizoma*. PPX and PPH are a CAS and hydroxyhopane synthase from *Polystichum polyblepharum*, respectively. Species of other clones are as follows: Pfi, *P. fibrilloso-paleaceum*; Ple, *P. lepidocaulon*; Plo, *P. longifrons*; Pma, *P. makinoi*; Pps, *P. pseudo-makinoi*; Pri, *P. rigens*.

The ORFs of the SC and OSC from these six ferns were successfully amplified. Primer sets were designed for the ORF amplification of PPH and PPX. The ORFs were ligated into the pT7 Blue T-Vector of a cloning vector, sequenced, and used to construct a neighbor-joining tree. In the fern CAS clade, the tree topology reflected chemotaxonomic classification except for PleCAS (*P. lepidocaulon* CAS, group 3), which was placed at the basal branch in the *Polystichum* subclade (Figure 5A). In the fern SC clade, the *Polystichum* SCs formed a polyphyletic group, including *Dryopteris* SC (DCD; Figure 5B). Those belonging to group 3 were grouped together. PriSC1 belonged to group 2 and was distantly related to the other two groups. However, the remaining three SCs, belonging to group 1, did not form a branch.

## 3. Discussion

### 3.1. Hydroxyhopane Synthases and Hopene Synthases

In the present study, a homological approach successfully isolated the SC cDNA sequence of PPH. Its functional expression in yeast revealed that PPH produced only 22-hydroxyhopane (5). This enzyme is the second reported example of a monofunctional hydroxyhopane synthase. PPH had the highest amino acid identity (90%) with ACH [11]. However, phylogenetic analysis showed that PPH was only distantly related to ACH despite the similarity of their product profiles. It was, in fact, closely related to *D. crassirhizoma* dammaradiene synthase (DCD; Figure 4) [12]. The fern SC clade in the phylogenetic tree reflects their taxonomic relationship rather than the similarity of their enzymatic functions.

**Figure 6:**
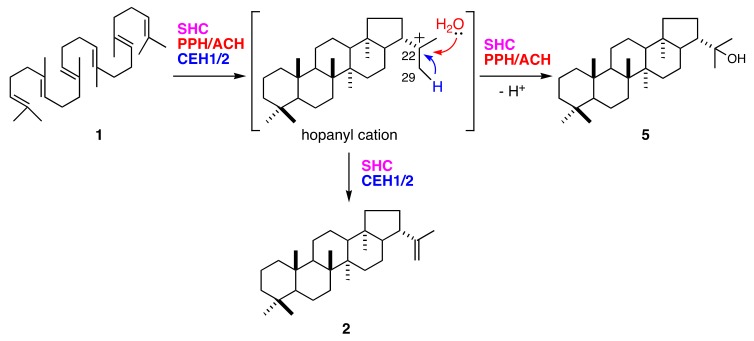
Cyclization of squalene (**1**) into hop-22(29)-ene (**2**) and 22-hydroxyhopane (**5**) by hopene and/or hydroxyhopane synthases.

Synthesis of 22-hydroxyhopane (**5**) consists of squalene (**1**) activation by cationic attack, followed by a cascade of cation-olefin cyclizations and quenching of the pentacyclic carbocation by the addition of water. The hop-22(29)-ene (**2**) reaction pathway is mostly shared by that of (**5**). They diverge at the final step in which their hopanyl cation quenching modes differ (Figure 6). Deprotonation of C29 from hopanyl cation produces (**2**). Bacterial hopene synthases (squalene-hopene cyclases; SHCs) also produce (**2**) and (**5**) [24,25]. The (**2**):(**5**) ratio is 84:16 for *Alicyclobacillus acidocaldarius* SHC [26]. In contrast, all fern hydroxyhopane- and hopene-synthases, including PPH, are monofunctional enzymes [11,14]. These findings suggest that fern hydroxyhopane- and hopene-synthases have more finely tuned catalytic capabilities than bacterial ones, with respect to the final cation elimination step.

The functions of 10 active site residues in AaSHC might not be strictly conserved in all fern SCs (Figure 3). Cys154 in PPH (and the corresponding Cys in ACH) might participate in the addition of water to the hopanyl cation. A previous study proposed that Trp133 in AaSHC is associated with the formation of a hydrogen-bonded water molecule network at the active site serving as a catalytic base [23]. The corresponding Tyr residue in CEH1 and CEH2 might have a function analogous to Trp with respect to aromaticity. In contrast, the Cys in PPH and ACH might not participate in the hydrogen-bonded network and could thus affect the water molecule as the catalytic base. Therefore, the final cation elimination reaction in PPH and ACH might favor the addition of water to the cation rather than its deprotonation.

Hoshino and colleagues proposed that Gln262 and Pro263 in AaSHC situate the catalytic base of the water molecule at its appropriate position, thereby contributing to the production of (**2**) and minimizing the formation of (**5**). However, these functions did not apply to fern SCs. Gln262 in AaSHC was replaced by Tyr in fern SCs (Figure 3). Therefore, Tyr was not responsible for the reaction selectivity of the catalytic base (deprotonation or water addition). Nevertheless, it might have, to some extent, contributed to the hydrogen bonding of water to the catalytic base. One of the active site residues, Pro263 in AaSHC, was identical to the corresponding residues in PPH and ACH. However, this identity does not explain the differences in the product profiles of AaSHC and PPH/ACH (Figure 3). In PPH and ACH, Pro263 might contribute to hydrogen bonding to the catalytic base, but does not influence product selectivity.

### 3.2. Relationship between Phylogeny and Taxonomy

Hopene synthases biosynthesize bacterial hopanoids. These compounds maintain membrane integrity by serving as sterol surrogates in several bacteria. Therefore, hopene synthases and/or hydroxyhopane synthases in ferns were speculated to be evolutionarily basal SCs. PPH from *P. polyblepharum* is a second hydroxyhopane synthase, assuming that PPH is clustered with the ACH of another hydroxyhopane synthase in the phylogenetic tree. However, a phylogenetic analysis based on the SC and OSC sequences revealed that PPH was only distantly related to ACH. The latter is the most basal in the fern SC clade (Figure 4). Therefore, we hypothesized that the fern SC clade reflects taxonomic relationships among ferns, but not the molecular evolutionary history of their enzyme functions. The phylogenetic tree in Figure 4 shows that PPH and DCD are clustered in the same branch despite the differences in their enzyme functions. This observation corroborates the hypothesis since *Polystichum* and *Dryopteris* both belong to Dryopteridaceae.

To test the hypothesis, we isolated cDNAs encoding SC and CAS from six *Polystichum* ferns. Since SC and CAS synthesize triterpenes, a phylogenetic tree reconstituted from these sequences could reflect the triterpene profile-based chemotaxonomic relationship. Therefore, the six chosen *Polystichum* plants had different triterpene profiles and were classified into three different groups (Figure 1). The phylogenetic analysis showed that *Polystichum* CASs were entirely separate from the other fern CASs (ACH and PnCAS). The phylogenetic tree topology of this subclade reflected chemotaxonomic classifications except for PleCAS (Figure 5A). A recent classification performed by Zhang and Barrington [27] arranged *Polystichum* into two subgenera whose identities were verified by molecular phylogenetic analysis with multiple plastid loci [28]. Of the seven species surveyed in the present study, *P. lepidocaulon* (PleCAS) belongs to the subgenus *Haplopolystichum*, whereas the others belong to the subgenus *Polystichum*. The relationship between PleCAS and the other six CASs implies that these two subgenera are distantly related. This finding might also be consistent with those reported for certain phylogenetic studies based on plastid markers. The clade consisting of the subgenus *Polystichum* appears to represent the chemotaxonomic relationship alone, rather than the previous classification supported by morphological features and conventional molecular data (plastid loci). Five of the six species belong to the *Hypopeltis* section, whereas *P. rigens* is a member of the *Xiphopolystichum* section. The fact that these two sections are grouped together suggests that the phylogenetic tree based on CASs and SCs might resolve relationships other than those revealed by plastid marker-based analyses.

In the fern SC clade, the phylogenetic tree topology seemed to correlate molecular phylogeny with chemotaxonomy despite the inclusion of DCD from *D. crassirhizoma* in the *Polystichum* subclade (Figure 5B). The member of group 3 and PpsSC1 in group 1 fell into the same subclade. PriSC1 in group 2 formed a branch with DCD and was clearly separated from the other groups. In contrast, no clear relationship among group 1 species was established. Unlike the CAS clade, each enzyme in the fern SC clade might have a different function. Enzyme function might influence the phylogenetic tree topology of the SC clade which, in turn, could reflect the combined chemotaxonomic classification and molecular evolution of SC functions. In that case, an SC subclade formed by a congener (*Polystichum*) could provide insight into the molecular evolution of the SC enzymes because the clade would no longer be influenced by taxonomical factors. The parameters responsible for the topology can be identified by characterizing the SCs isolated in the present work. Further experiments are also required to validate the hypothesis that SC- and/or OSC-based phylogenetic trees reflect triterpene profile-based chemotaxonomic classification.

The present study showed that the primer set designed for PPH and PPX enabled the isolation of SC and OSC genes from the other six congeners. This approach might contribute to the rapid isolation of SC-encoding genes from other taxa in the future. To our knowledge, this is also the first study to focus on the comparison between the triterpene profile-based chemotaxonomy and conventional molecular phylogeny: Both of which were connected to each other, by using the sequences encoding SC and OSC. The present phylogenetic analysis showed that the triterpene profile might be related to the corresponding gene sequences. However, this interpretation might be altered because the physiological roles of triterpenes were not considered in the present study. The function of metabolites apparently affects the evolutionary history of organisms. Nonetheless, drawing any conclusive answer to the intriguing question of the relationship between chemistry and phylogeny is still difficult. Nevertheless, the application of the present approach to other fern genera is expected to provide both a deeper and more general understanding of triterpene biosynthesis and chemotaxonomic classification in ferns.

## 4. Materials and Methods

### 4.1. RNA Extraction and cDNA Synthesis

*Polystichum polyblepharum*, *P. fibrillosopaleaceum*, *P. pseudo-makinoi*, *P. rigens*, *P. longifrons*, *P. makinoi*, and *P. lepidocaulon* were propagated in the Medicinal Plant Garden of Showa Pharmaceutical University. Their fronds were harvested for total RNA extraction, which was performed using the RNeasy Plant Mini Kit (Qiagen, Hilden, Germany) according to manufacturer’s instruction. First-strand cDNA was synthesized from the RNA by using SuperScript III reverse transcriptase (Thermo Fisher Scientific, Waltham, MA, USA) and an oligo (dT) primer (RACE 32 [20], 5′-GACTCGAGTCGACATCGATTTTTTTTTTTTTT-3′) according to manufacturer’s instruction. For 3′- and 5′-RACE, an adaptor-ligated double-strand cDNA was synthesized using a SMARTer RACE 5′/3′ Kit (TaKaRa Bio, Shiga, Japan) according to manufacturer’s instruction.

### 4.2. Cloning of Squalene Cyclase cDNAs

Homology-based nested PCR was performed using the single-strand cDNA and five degenerate primers [11,12] (SC-306S-1, SC-306S-2, SC-358S, SC-494A, and SC-537A). PCR was performed using Ex *Taq* DNA polymerase Hot Start Version (TaKaRa Bio, Shiga, Japan) in a final volume of 50 μL PCR conditions were identical to those reported in our previous study [11]. The primer-specific amplicon was purified from an agarose gel, cloned into pT7 Blue T-Vector (Merck KGaA, Darmstadt, Germany), and transformed into *E. coli* strain DH5α. Plasmid DNA was purified from transformed cells by using an Illustra PlasmidPrep Mini Spin Kit (GE Healthcare, Chicago, IL, USA) and sequenced using a BigDye terminator cycle sequence kit v.3.1 (Thermo Fisher Scientific, Waltham, MA, USA) and an ABI PRISM 3130 genetic analyzer (Thermo Fisher Scientific, Waltham, MA, USA). The sequence was used to design two specific primers (PPH-481A for first PCR, 5′-TTGCTCTCTCCTGAGATATGTCAAG-3′; PPH-468A for second PCR, 5′-ATTCCGGGGGCAGCACATTGGCCTC-3′). Nested PCR was run using the specific primers, SC306S-1, and the same PCR conditions as described above. The PCR product was purified, subcloned, and sequenced.

Based on the sequenced fragment, we designed gene-specific primers for 5′- and 3′-RACE: For 5′-RACE, PPH-451A for first PCR, 5′-AAGCTAGTACCTTGCAGTTTCAACCTG-3′; PPH-345A for second PCR, 5′-TGAATGACCAATCTCCATGCTTTGTGA-3′; for 3′-RACE, PPH-414S for first PCR, 5′-ATGGATATCGTCTATGCAGGCCAGAGG-3′; PPH-461S for second PCR, 5′-CTGCAAGGTACTAGCTTTGATGAGG-3′. RACE PCR was performed using the adaptor-ligated double-strand cDNA as a template and gene-specific primers by using SMARTer RACE 5′/3′ Kit (TaKaRa Bio, Shiga, Japan) according to manufacturer’s instruction. The full-length nucleotide sequence was named PPH.

To isolate the ORF of the SC from the six *Polystichum* ferns, we performed PCR by using PPH-specific primers designed for ORF amplification (N-PPH and C-PPH described below) and single-strand cDNA as a template. The first PCR was performed using KOD-Plus v. 2 (Toyobo, Osaka, Japan) in a final volume of 50 μL. PCR conditions were identical to those reported in our previous study [13]. The second PCR was the same as the first except the first PCR product was a template for putative SC-encoding cDNAs designated PfiSC1 from *P. fibrillosopaleaceum*, PpsSC1 from *P. pseudo-makinoi*, PriSC1 from *P. rigens*, PloSC1 from *P. longifrons*, PmaSC1 from *P. makinoi*, and PleSC1 from *P. lepidocaulon*. The primer-specific amplicon was purified on agarose gel, cloned into pT7 Blue T-Vector (Merck KGaA, Darmstadt, Germany), and transformed into *E. coli* strain DH5α. Plasmid DNA was purified from transformed cells and sequenced.

The seven nucleotide sequences were deposited in GenBank/EMBL/DDBJ under accession numbers LC389069 for PPH, LC389071 for PloSC1, LC389072 for PfiSC1, LC389073 for PmaSC1, LC389074 for PriSC1, LC389075 for PleSC1, and LC389076 for PpsSC1.

### 4.3. PPH Expression in Pichia pastoris and Product Analysis

The coding region of PPH was amplified using nested PCR by using primers (5′-PPH, 5′-CGCCCGGGCAGGTATTGATGTTAGG-3′ and 3′-PPH, 5′-AGGCTGCTTGCTATGAAGCTTGCAG-3′ for the first PCR; N-PPH, 5′-TTAGGCCTCGAGATGCTGCCATACAACCAAGAT-3′, and C-PPH, 5′-CAAGGCGCGGCCGCTTATGGAATTGGAGGCTTGAT-3′. The method used was the same as that described in our previous study [11]. After treatment with XhoI and NotI, the PCR product was inserted into the corresponding pPIC6B restriction sites (Thermo Fisher Scientific, Waltham, MA, USA). *Pichia pastoris* strain X-33 was transformed using the resultant plasmid by using the method reported in our previous study [13].

The transformed *P. pastoris* was grown in yeast extract peptone dextrose (YPD) medium and induced using methanol in glucose-free YPD medium. The cells were resuspended in potassium phosphate (0.1 M, pH 7.0) supplemented with glucose and terbinafine [29]. They were saponified with ethanolic KOH and extracted with *n*-hexane. The culture conditions and process were the same as those reported in our previous study [14].

The hexane extract was passed through a BondElut-Si column (500 mg, 3 mL; Varian, Santa Clara, CA, USA) and eluted with *n*-hexane/ethyl acetate (9:1, *v*/*v*) to yield the triterpene hydrocarbon and triterpene mono-alcohol fractions (Fraction-A). Fraction A was analyzed using gas chromatography on a Hitachi G-6000 instrument (Hitachi Hi-Technologies, Tokyo, Japan) equipped with a DB-5HT column (30 m × 0.25 mm; Agilent Technologies, Santa, Clara, CA, USA) under the same conditions as those reported in our previous study [13]. A PPH product was purified from Fraction A by using a BondElut-Si column (500 mg, 3 mL; Varian, Santa Clara, CA, USA) and eluted with a 0–3% ethyl acetate gradient in *n*-hexane. The ^1^H NMR spectrum of the purified product was measured in CDCl_3_ (Bruker AV600; Billerica, MA, USA), by using tetramethylsilane as an internal standard.

22-Hydroxyhopane (**5**): ^1^H NMR (600 MHz, CDCl_3_): δ 0.76 (s, 3H), 0.79 (s, 3H), 0.81 (s, 3H), 0.85 (s, 3H), 0.96 (s, 6H), 1.18 (s, 3H), 1.21 (s, 3H).

### 4.4. Cloning of Oxidosqualene Cyclase cDNA

A homology-based nested PCR was performed using the single-strand cDNA and five degenerate primers [20,21] (OSC-162S, OSC-463S, OSC-467S, OSC-623A, and OSC-711A). The PCR, subcloning, and sequencing were the same as those described above. The sequence was used to design two specific primers (PPX-504A for the first PCR, 5′-TTGTATTCACTGCATCGTAGAAGCG-3′; PPX-482A for the second PCR, 5′-GACTCAACGCTAGTGCAGCCTTAAA-3′). Nested PCR was performed using the specific primers and OSC-162S. PCR conditions were the same as those described above. The PCR product was purified, subcloned, and sequenced.

Based on the sequenced fragment, we designed gene-specific primers for 5′- and 3′-RACE: For 5′-RACE, PPX-206A for the first PCR, 5′-TTTTCCCCATGAGGGAATGGCTGTAGC-3′; PPX-189A for the second PCR, 5′-TCTTCCTCTCTCCATGGCTTGATCCAC-3′; for 3′-RACE, PPX-566S for the first PCR, 5′-AGTCATCCAAGGCTTAGCAGCCTTC-3′; PPX-588S for the second PCR, 5′-ATGCATTGAGCGTGCTGCTTGCTAC-3′. RACE PCR was performed using a SMARTer RACE 5′/3′ Kit (TaKaRa Bio, Shiga, Japan) according to manufacturer’s instruction by using the adaptor-ligated double-strand cDNA as a template and gene-specific primers.

To isolate the ORF of SC from the six *Polystichum* ferns, we performed PCR by using PPX-specific primers designed for ORF amplification (N-PPX and C-PPX described below) and single-strand cDNA as a template. The PCR conditions, plasmid construction, and sequencing were the same as those described in Section 4.2. Putative CAS-encoding cDNAs were designated PfiCAS from *P. fibrillosopaleaceum*, PpsCAS from *P. pseudo-makinoi*, PriCAS from *P. rigens*, PloCAS from *P. longifrons*, PmaCAS from *P. makinoi*, and PleCAS from *P. lepidocaulon*.

The seven nucleotide sequences were deposited in GenBank/EMBL/DDBJ under accession number LC389070 for PPX, LC389077 for PloCAS, LC389078 for PfiCAS, LC389079 for PmaCAS, LC389080 for PriCAS, LC389081 for PleCAS, and LC389082 for PpsCAS.

### 4.5. Expression of PPX in Pichia pastoris and Analysis of Product

The coding region of PPX was amplified using nested PCR by using primers (5′-PPX, 5′-ACCAAAAGCGTGTAGAGAGAGAGAG-3′ and 3′-PPX, 5′-AGCTTGCCAACAATGATGCTGGATG-3′ for the first PCR; N-PPX, 5′-GAGAGAGAATTCGAAATGTGGAGCTTGAAGACAGCA-3′, and C-PPX, 5′-CACCTAGCGGCCGCTCAATGATGATGATGATGATGTTTATAGCTCAAAACGCTTCG-3′ for the second PCR) by using the same method as that described above. After treatment with EcoRI and NotI, the PCR product was inserted into the corresponding pPIC3.5 restriction sites (Thermo Fisher Scientific, Waltham, MA, USA). *Pichia pastoris* strain GS115 was transformed with the resultant plasmid by using the same method as that described above.

The transformed *P. pastoris* was grown in 25 mL minimal glycerol medium (1.34% *w*/*v* yeast nitrogen base with ammonium sulfate and no amino acids (YNB), 1% *w*/*v* glycerol, 4 × 10^−5^ biotin) at 30 °C for 24 h with shaking. The cells were then induced in 100 mL minimal methanol medium (1.34% YNB, 0.5% *v*/*v* methanol, 4 × 10^−5^ biotin) at 30 °C for 24 h with shaking. They were suspended in 10 mL extraction buffer [50 mM sodium phosphate (pH 7.4); 1 mM dithiothreitol, 5% *w*/*v* glycerol, and 0.8% *w*/*v* Protease Inhibitor Cocktail (Merck KGaA, Darmstadt, Germany)]. Glass beads (ø 0.35–0.5 mm, 5 mL) were added to the suspension, followed by 10 cycles of vortexing for 30 s cycle^−1^ at 30-s intervals and 4 °C. After cell disruption, Triton X-100 (final concentration, 0.2% *w*/*v*) was added to the homogenate. It was then centrifuged to separate the glass beads and cellular debris. The resulting supernatant was used as crude enzyme in the following procedure.

The crude enzyme solution (2.5 mL) was incubated at 30 °C for 20 h, with 250 μg (3*RS*)-oxidosqualene (**10**) in a total volume of 5 mL containing: 50 mM sodium phosphate buffer (pH 7.4), 0.2% *w*/*v* Triton X-100, and 1 mM dithiothreitol. The reaction was stopped by refluxing it with 5 mL of 20% *w*/*v* KOH in 50% *v*/*v* EtOH. After two extractions with 10 mL *n*-hexane, the organic layer was concentrated. The hexane extract was subjected to BondElut-Si (500 mg, 3 mL; Varian, Santa Clara, CA, USA) and eluted with *n*-hexane/ethyl acetate (4:1, *v*/*v*) to yield a triterpene mono-alcohol fraction (Fraction B). Fraction B was analyzed using gas chromatography performed on a Hitachi G-6000 instrument (Hitachi Hi-Technologies, Tokyo, Japan) equipped with a DB-5HT column (30 m × 0.25 mm; Agilent Technologies, Santa Clara, CA, USA) under the same conditions as those described above. Substrate **10** was chemically synthesized according to the literature [30].

### 4.6. Phylogenetic Analysis

The deduced amino acid sequences of PPH and PPX were phylogenetically analyzed against the SC and OSC sequences from plants, bacteria, fungi, and mammals as well as 90 amino acid sequences retrieved from the GenBank/EMBL/DDBJ database. The SC and OSC sequences are shown in Supplementary Table S1. The sequences were aligned using CLUSTAL W [31] by using default parameters. The evolutionary history was inferred using the neighbor-joining method [32]. The phylogenetic tree is drawn to scale. Its branch lengths are in the same units as those of the evolutionary distances used to infer the phylogenetic tree itself. The evolutionary distances were computed using the Poisson correction method [33] and are expressed as the number of amino acid substitutions per site. The estimated reliability of the phylogenetic tree was tested using the bootstrap method (500 replications) [34]. Evolutionary analyses were conducted in MEGA7 [35].

Phylogenetic analysis was also conducted for the dataset, including the additional six *Polystichum* SCs (PfiSC1, PpsSC1, PriSC1, PloSC1, PmaSC1, and PleSC1), the six OSCs (PfiCAS, PpsCAS, PriCAS, PloCAS, PmaCAS, and PleCAS), and the above 92 sequences. The methods and conditions used for sequence alignment and phylogenetic inference were the same as those described above.

## Supplementary Materials

The supplementary materials are available online; Figure S1: Phylogenetic tree of triterpene synthases, Figure S2: Amino acid sequence alignment of PPH and hydroxyhopane and hopene synthases from ferns and a bacterium, Figure S3: Phylogenetic tree of CAS clade (A) and fern SC clade (B), Table S1: Sequence information used in the phylogenetic analysis.
