# Supplementary material for: Squalene Cyclases and Cycloartenol Synthases from Polystichum polyblepharum and Six Allied Ferns
Source: Molecules. 2018 Jul 24;23(8):1843. doi: 10.3390/molecules23081843 (PMC6222715; doi:10.3390/molecules23081843)
Supplement: Supplementary file 1 [file molecules-23-01843-s001.pdf]

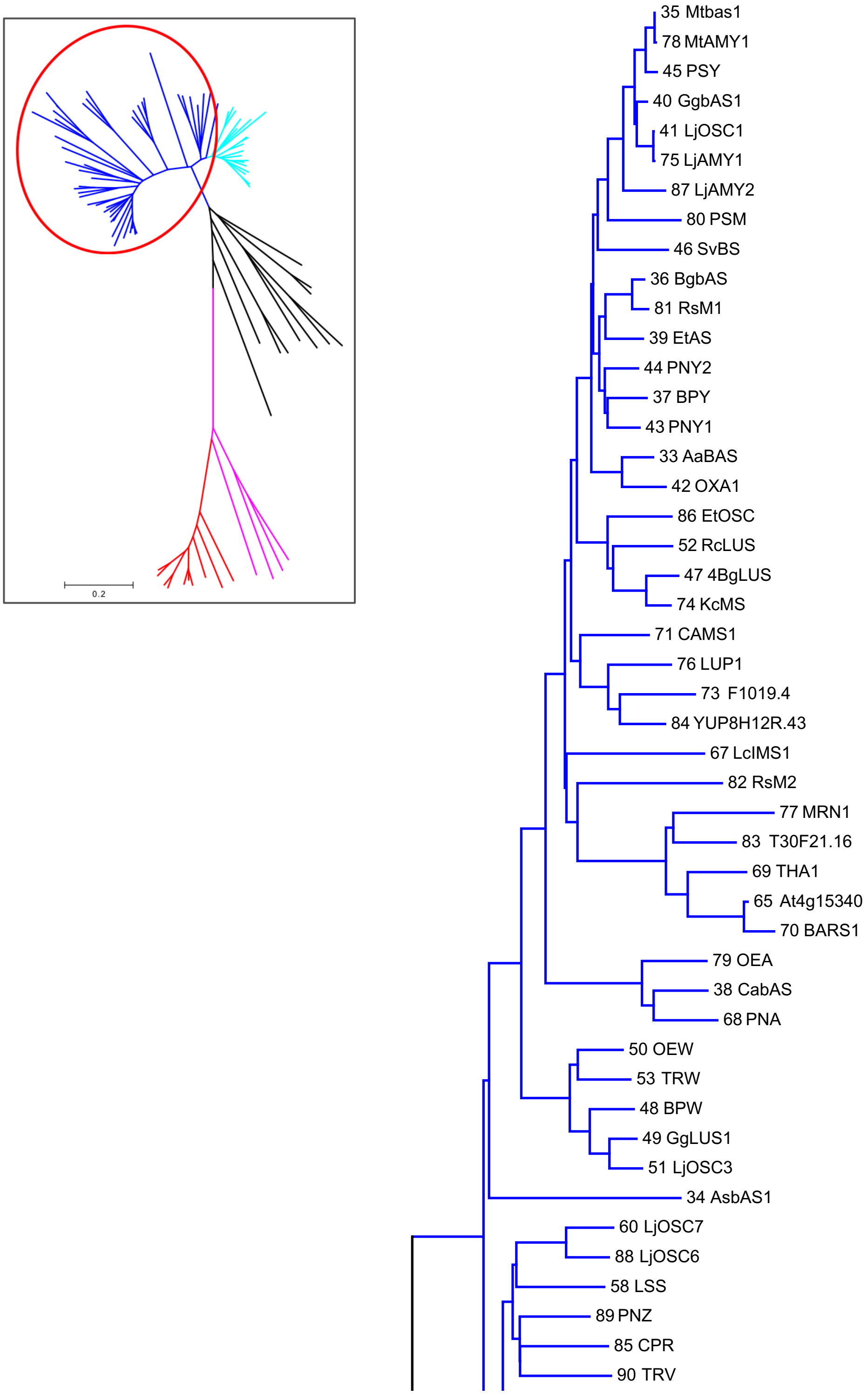

Supplementary Figure 1. Phylogenetic tree of triterpene synthases. Inlet tree shows Figure 1A. Red circle indicates expanded region.

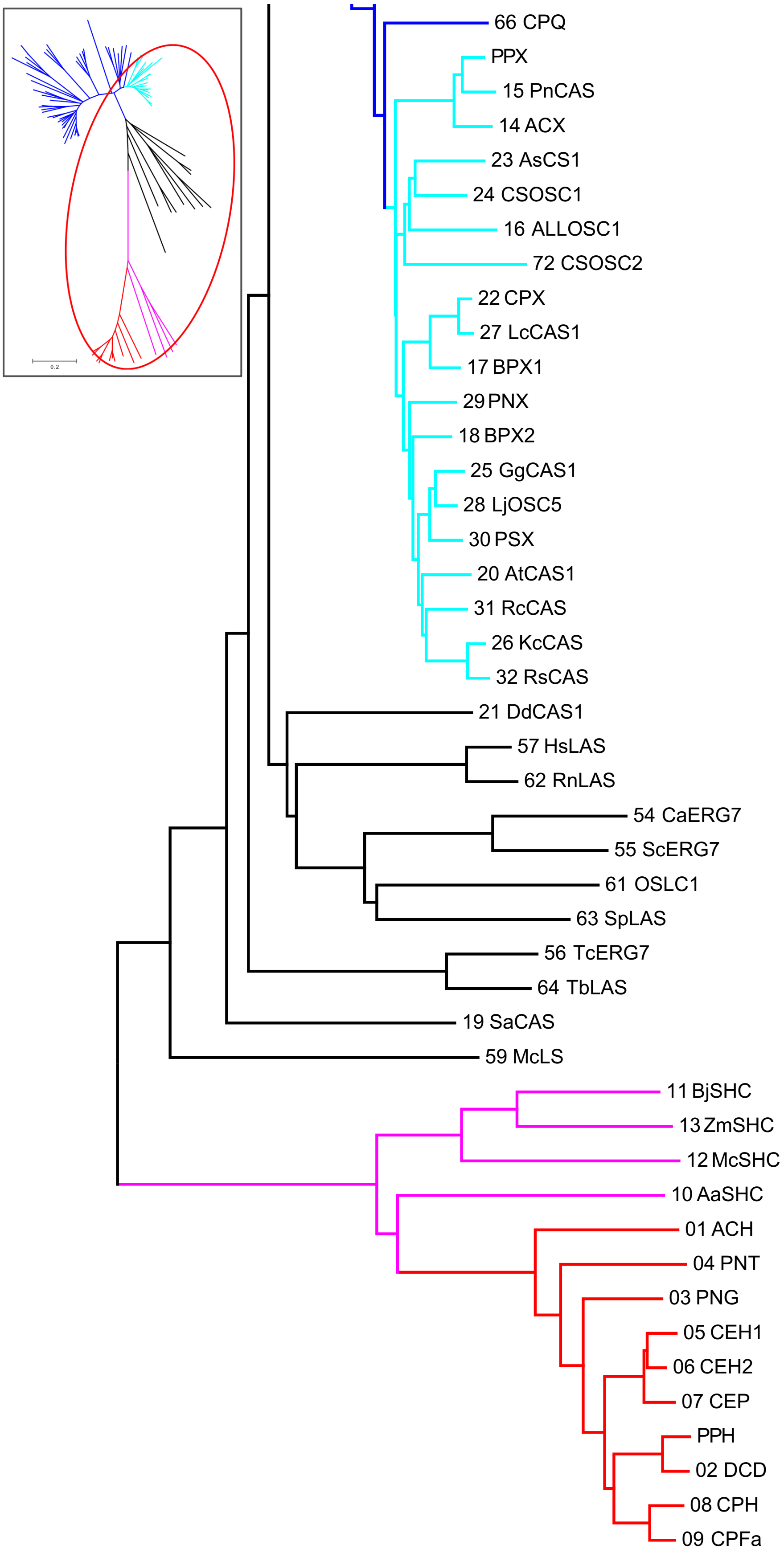

Supplementary Figure 1. Continued.

```
PPH      1 M-LPYNQDF----YNEDEAIKDGHSKGAGNVTTPPTLEEAIKSSQDFLLSQQYPEGYWWA
ACH      1 M-LPYNQDHH---FG----------KVAENATMPPTLDEAIERSQDFLLSLQYPEGYWWA
CEH1     1 MALPYNQDLQNNIFAGDEAA-DDCKLGAADSRPIPSVDEAIKRTQNFLLGQQFPEGYWWG
CEH2     1 MALPYNQDLQNNIFAGDEAA-DDCKLGATDSRPVPSVDEAIKRTQNFLLGQQFPEGYWWG
AaSHC    1 MA--------------------------EQLVEAPAYARTLDRAVEYLLSCQKDEGYWWG

PPH     56 ELEANATITSHTVILYKILGIEDECPMDKMEKYLRRMQCSHGGWELFYGDGGALSVTIES
ACH     47 ELEANVTLTAQTIMLYKILGIDHKYPIHKMKTYILRTQRAHGGWEIFYGDGGCLSTTIGA
CEH1    60 EFESNVTVATHTVLLYKILGIEEKYPMHKFERYLRRTQCSHGGWELSYGDGGYLSATIEA
CEH2    60 EFEGNVTVATHTVLLYKILGIEEKYPMHKFERYLRRKQCSHGGWEISYGDGGYLSATIEA
AaSHC   35 PLLSNVTMEAEYVLLCHILDRVDRDRMEKIRRYLLHEQREDGTWALYPGGPPDLDTTIEA

PPH    116 YIALRLLNVPRTDPALKTALKFIVDKGGVTKSRMFTKICLALLGCFDWRSIPSLPPWVML
ACH    107 YMALRILGVPKTDPVLQKALKLIHSKGGVTKSRMFTKICLALLGCYDWKGIPSLPPWLVL
CEH1   120 YIALRLLNVPRSDPALTKALQVILDGGGVTKARIFTKLYLALLGCFDWTGLPSLPPWLIL
CEH2   120 YIALRLLNVPRSDPALTKALQVILDGGGVTKARIFTKLYLALLGCFDWTGLPSLPPWLIL
AaSHC   95 YVALKYIGMSRDEEPMQKALRFIQSQGGIESSRVFTRMWLALVGEYPWEKVPMVPPEIMF

PPH    176 LPSWFLSSIYETACWARGCVVPLIVVFDKKPVFKVSPEVSFDELYAEGREHACKTLPF-C
ACH    167 LPSWFPFSLYDTASWVRGCVVPLTIIFDKKPVYKLNPLLCLDELYSEGKGKARVHLSFIP
CEH1   180 FPAWFLSSIYEINCPTRGCVVPLTVVLDKKPVFKVSPEVSFDELYVEGRAHACKALPF-S
CEH2   180 FPAWFLSSIYETACWARASVVPLTVVLDKKPVFKVSPEVSFDELYTEGRGHACKALPF-S
AaSHC  155 LGKRMPLNIYEFGSWARATVVALSIVMSRQPVFPLPERARVPELY-ETDVPPRRRGAKGG

PPH    235 GHLTSDFFIAVDHVFKMMERLGVVPFRQWGIREAEKWLLERQE-DTGDYSGAYPPMFYSV
ACH    227 GDWTSNFFVGLDHVFKYMENLGVVPFRQWGIKEAERWTLERHE-DSGDFHGIYPPMFYSI
CEH1   239 GDWTSKLFITVDRILKMMERFGVVPFRQRSFREAEKWLLERLEPESGQFVGAYAPMFYSV
CEH2   239 GDWTSKLFIMVDRILKMMDRFGVVPFRQRCFREAEKWLLERLEPESGELVGVYAPMFYSV
AaSHC  214 GGW---IFDALDRALHGYQKLSVHPFRRAAEIRALDWLLER-QAGDGSWGGIQPPWFYAL

PPH    294 VCMKTLGYQVTDPVVQRALLAFKNFSIERADECSV-QSTLSPVWDTALVVRSLVESGLPP
ACH    286 VSYSLLGYEITDPVVHRALESMRGFTVEREDECVV-QSCISPMWDTAFVIRSLAESGLQP
CEH1   299 VCMKILGYEVTDPVVQRALLGFKLFTIETADECWV-QATISPVWDTAFIVRALVESGIPP
CEH2   299 VCMKTLGYEVTDPVVQRALLGFKLFTIETADECWV-QASISPVWDTAFIVRALVESGIPP
AaSHC  270 IALKILD-MTQHPAFIKGWEGLELYGVELDYGGWMFQASISPVWDTGLAVLALRAAGLPA

PPH    353 DHPALQKAGEWLIQKQITKHGDWSFKNPSG-VAGGWAFQFFNRWYPDLDDSAVVVMALDC
ACH    345 DHPALQKAGEWLLQKQATQHGNWFYKKRTGR-AGGWAFQFFNRWYPDVDDSAAVSMALNA
CEH1   358 DHPALQKAGQWLLRKQILKHGDWTFKAGSGHLAGGWAFQFYNGWYPDIDDSAAVVMALDC
CEH2   358 DHPALQKAGQWLLQKQILKHGDWTFKAGSRHLAGGWAFQFYNRWYPDADDSAAVVMALDC
AaSHC  329 DHDRLVKAGEWLLDRQITVPGDWAVKRPNLK-PGGFAFQFDNVYYPDVDDTAVVVWALNT
```

Supplementary Figure 2. Amino acid sequence alignment of PPH and hydroxyhopane and hopene synthases from ferns and a bacterium. Conserved residues are highlighted in pale blue. Active site residues responsible for the final deprotonation reaction are highlighted in red. ACH, hydroxyhopane synthase from *Adiantum capillus-veneris*; CEH1 and CEH2, hopene synthases from *Colysis elliptica*; AaSHC, hopene synthase from *Alicyclobacillus acidocaldarius*.

```
PPH     412 LKLPNEDVKNGAITRCLRWISSMQARGGGWAAYDKDSHQHWINSTPFSDLKAMLDPSTAD
ACH     404 IKLQDDDVKKGAIKRCAEWISVMQCKDGGWAAYDCDNDREWLNCTPFGDLKAMIDPNTVD
CEH1    418 IKLPDEDVKNGAIARCLKWLSIMQSRSGGWAAFDKNAEQHWIDSTPFSDIKAMLDSSSSD
CEH2    418 IKLPDEDVKNGAMARCLKWLSIIQSRSGGWASFDKNADQHWIDSIPFSDIKGMLDSSTSD
AaSHC   388 LRLPDERRRRDAMTKGFRWIVGMQSSNGGWGAYDVDNTSDLPNHIPFCDFGEVTDDPPSED

PPH     472 VTARVLEMVGRLKLQ----GTSFDEAN-----VLPPESIARGLTYLRREQENEGCWFGRW
ACH     464 VTARVLEMVGRVK------------EAGDASAILPPRAIARGLAYLRREQETEGCWYGRW
CEH1    478 ISARVLEMVGRLRLDLSAAGTPPARQRSALVALMPPEAIARGLAYLRRDQEEEGCWWGRW
CEH2    478 VSARVLEMVGRLKLDLSGAGTPLAKQRGALVALMPPEAIARGLAYLRREQEEEGCWWGRW
AaSHC   448 VTAHVLECFGSF---------GYDDAW---------KVIRRAVEYLKREQKPDGSWFGRW

PPH     523 GVNYIYGTCGDLVALSLVAPMAHAEEIARGARWLVQVQNMHG------------------
ACH     512 GVNYIYGTSGALMALALVAPSTHKEEIERGARWLVEVQNKRGTKGANGYSHTNGAREGGV
CEH1    538 GVNYIYGTCGALIALSLVAPTTHEEEIARGAKWLVQVQNRLGNN----------------
CEH2    538 GVNYIYGTCGALMALSLVAPTTHEEEIARGAKWLVQVQNRLGNN----------------
AaSHC   490 GVNYLYGTGAVVSALKAVGIDTREPYIQKALDWVEQHQNP--------------------

PPH     565 ------KKINGPQDGGWGETCFSYNDPALRGQGDVSTASQTAWALQGLLAAGDALGKYEV
ACH     572 AMNGNCKNMGAPEDGGWGETCFSYNDITLKGRNEVSTVSQTAWALQGLLAAGDALGKYEV
CEH1    582 ------KVLHGPQDGGWGETCLSYNDPALKAQSDASTASQTAWALQGLLAAGDALGKYEV
CEH2    582 ------KVLHGPQDGGWGETCLSYNDPALKEQSDASTASQTAWALQGLLAAGDALGKYEV
AaSHC   530 -------------DGGWGEDCRSYEDPAYAGKG-ASTPSQTAWALMALIAGGRA----ES

PPH     619 ESIEQGVQYLLPTQRKDGSWHEPHFTGGGFPIHFYLRYHFYPQHFTLGFLARYRTRMEAS
ACH     632 ESIEHGVQYLLSTQRKDGSWCEKHFTGGGFPRFFYIRYHLYAGHFPLSALARYRDRVRAG
CEH1    636 EAMEQGVQYLLATQRKDGSWHESQFTGCGSPIHFYFRYHYYAQHFPLSALARYRTRILQV
CEH2    636 EAMEQGVQYLLATQRKDGSWHESQFTACGFPMHFYLKYHYYAQHFPLSALARYRTRILQA
AaSHC   572 EAARRGVQYLVETQRPDGGWDEPYYTGTGFPGDFYLGYTMYRHVFPTLALGRYKQAIERR

PPH     679 KIKPPIP
ACH     692 KMAK
CEH1    696 SHTN
CEH2    696 SHTN
```

Supplementary Figure 2. Continued.

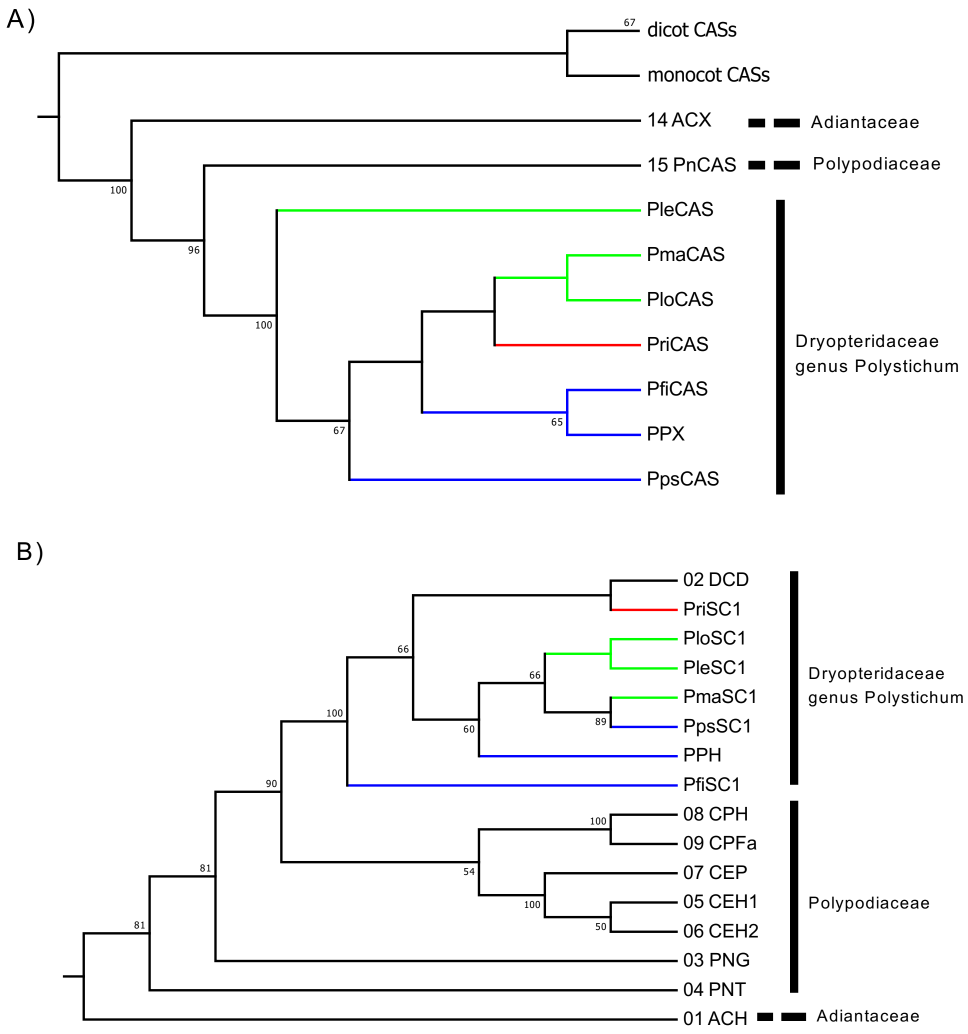

Supplementary Figure 3. Phylogenetic tree of CAS clade (A) and fern SC clade (B).

Supplementary Table 1. Sequence information used in the phylogenetic analysis

	Accession No.	Clone	Origin	Function
1	AB368376	ACH	*Adiantum capillus-veneris*	hydroxyhopane
2	AB429303	DCD	*Dryopteris crassirhizoma*	dammaradiene
3	AB530327	PNG	*Polypodiodes niponica*	germanicene
4	AB530326	PNT	*Polypodiodes niponica*	tirucalladiene
5	LC074708	CEH1	*Colysis elliptica*	hop-22(29)-ene
6	LC074709	CEH2	*Colysis elliptica*	hop-22(29)-ene
7	LC074710	CEP	*Colysis elliptica*	a-polypodatetraene
8	LC074711	CPH	*Colysis pothifolia*	hop-22(29)-ene
9	LC074712	CPFa	*Colysis pothifolia*	fern-9(11)-ene
10	M73834	AaSHC	*Alicyclobacillus acidocaldarius*	hop-22(29)-ene
11	X86552	BjSHC	*Bradyrhizobium japonicum*	
12	Y09978	McSHC	*Methylococcus capsulatus*	
13	X80766	ZmSHC	*Zymomonas mobilis*	
14	AB368375	ACX	*Adiantum capillus-veneris*	CAS
15	AB530328	PnCAS	*Polypodiodes niponica*	
16	AB025353	ALLOSC1	*Allium macrostemon*	
17	AB055509	BPX1	*Betula platyphylla*	
18	AB055510	BPX2	*Betula platyphylla*	
19	AJ494839	SaCAS	*Stigmatella aurantiaca*	
20	U02555	AtCAS1	*Arabidopsis thaliana*	
21	AF159949	DdCAS1	*Dictyostelium discoideum*	
22	AB116237	CPX	*Cucurbita pepo*	
23	AJ311790	AsCS1	*Avena strigosa*	
24	AB058507	CSOSC1	*Costus speciosus*	
25	AB025968	GgCAS1	*Glycyrrhiza glabra*	
26	AB292609	KcCAS	*Kandelia candel*	
27	AB033334	LcCAS1	*Luffa cylindrica*	
28	AB181246	LjOSC5	*Lotus japonicus*	
29	AB009029	PNX	*Panax ginseng*	
30	D89619	PSX	*Pisum sativum*	
31	DQ268870	RcCAS	*Ricinus communis*	
32	AB292608	RsCAS	*Rhizophora stylosa*	

Supplementary Table 1. continued

	Accession No.	Clone	Origin	Function
33	EU330197	AaBAS	*Artemisia annua*	bAS
34	AJ311789	AsbAS1	*Avena strigosa*	
35	AJ430607	Mtbas1	*Medicago truncatula*	
36	AB289585	BgbAS	*Bruguiera gymnorrhiza*	
37	AB055512	BPY	*Betula platyphylla*	
38	AY520818	CabAS	*Centella asiatica*	
39	AB206469	EtAS	*Euphorbia tirucalli*	
40	AB037203	GgbAS1	*Glycyrrhiza glabra*	
41	AB181244	LjOSC1	*Lotus japonicus*	
42	AY836006	OXA1	*Aster sedifolius*	
43	AB009030	PNY1	*Panax ginseng*	
44	AB014057	PNY2	*Panax ginseng*	
45	AB034802	PSY	*Pisum sativum*	
46	DQ915167	SvBS	*Vaccaria hispanica*	
47	AB289586	BgLUS	*Bruguiera gymnorrhiza*	LUS
48	AB055511	BPW	*Betula platyphylla*	
49	AB116228	GgLUS1	*Glycyrrhiza glabra*	
50	AB025343	OEW	*Olea europaea*	
51	AB181245	LjOSC3	*Lotus japonicus*	
52	DQ268869	RcLUS	*Ricinus communis*	
53	AB025345	TRW	*Taraxacum officinale*	
54	L04305	CaERG7	*Candida albicans*	LAS
55	U04841	ScERG7	*Saccharomyces cerevisiae*	
56	AF285826	TcERG7	*Trypanosoma cruzi*	
57	D63807	HsLAS	*Homo sapiens*	
58	AB247155	LSS	*Arabidopsis thaliana*	
59	AE017282	McLS	*Methylococcus capsulatus*	
60	AB244671	LjOSC7	*Lotus japonicus*	
61	AF327881	OSLC1	*Cephalosporium caerulens*	
62	D45252	RnLAS	*Rattus norvegicus*	
63	U41368	SpLAS	*Schizosaccharomyces pombe*	
64	AF226705	TbLAS	*Trypanosoma brucei brucei*	
65	AB257562	At4g15340	*Arabidopsis thaliana*	arabidiol

Supplementary Table 1. continued

	Accession No.	Clone	Origin	Function
66	AB116238	CPQ	*Cucurbita pepo*	cucurbitadienol
67	AB058643	LcIMS1	*Luffa cylindrica*	isomultiflorenol
68	AB265170	PNA	*Panax ginseng*	dammarenediol
69	AB017064	THA1	*Arabidopsis thaliana*	thalianol
70	AL161541	BARS1	*Arabidopsis thaliana*	multifunctional
71	AC002986	CAMS1	*Arabidopsis thaliana*	
72	AB058508	CSOSC2	*Costus speciosus*	
73	AF489920	F1019.4	*Arabidopsis thaliana*	
74	AB257507	KcMS	*Kandelia candel*	
75	AF478454	LjAMY1	*Lotus japonicus*	
76	U49919	LUP1	*Arabidopsis thaliana*	
77	AB013391	MRN1	*Arabidopsis thaliana*	
78	AF478453	MtAMY1	*Medicago truncatula*	
79	AB291240	OEA	*Olea europaea*	
80	AB034803	PSM	*Pisum sativum*	
81	AB263203	RsM1	*Rhizophora stylosa*	
82	AB263204	RsM2	*Rhizophora stylosa*	
83	AC007260	T30F21.16	*Arabidopsis thaliana*	
84	AY091131	YUP8H12R.43	*Arabidopsis thaliana*	
85	AB116239	CPR	*Cucurbita pepo*	unknown
86	AB206470	EtOSC	*Euphorbia tirucalli*	
87	AF478455	LjAMY2	*Lotus japonicus*	
88	AB244670	LjOSC6	*Lotus japonicus*	
89	AB009031	PNZ	*Panax ginseng*	
90	AB025346	TRV	*Taraxacum officinale*	
